# Commentary: Blended learning in physical education: a systematic review

**DOI:** 10.3389/fpubh.2025.1549858

**Published:** 2025-03-26

**Authors:** Yuan Li, Gubao Xu

**Affiliations:** ^1^Faculty of Health Sciences and Sports, Macao Polytechnic University, Macao, Macao SAR, China; ^2^Department of Physical Education, Xi'an University of Posts and Telecommunications, Xi'an, Shaanxi, China

**Keywords:** blended learning, physical education, educational technology, learning motivation, teaching challenge

Wang et al. (1) in *Blended Learning in Physical Education: In A Systematic Review*, 22 articles from the Web of' Science database were reviewed to systematically summarize the research progress of blended learning in physical education. The authors analyzed the application of blended learning in physical education in detail from multiple dimensions, such as research trends, participant groups, learning tools, and evaluation methods, and suggested future research directions. The purpose of this commentary is to critically comment on the main findings of the study, particularly the limitations of the study design, theoretical framework, and evaluation methods, and to discuss the potential impact of these limitations on the conclusions of the study.

**Figure 1:**
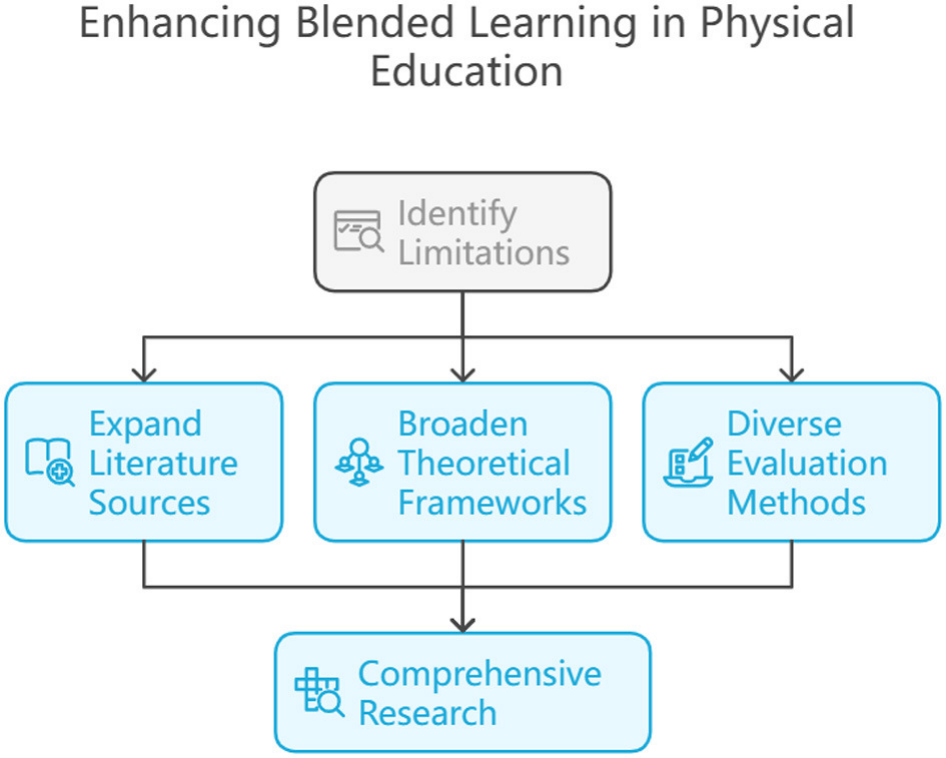
Theoretic inference and suggestions.

Page et al. (2) used the Preferred Reporting Items for Systematic Reviews and Meta-analyses (PRISMA) method to screen 22 literatures, which ensured the quality of literatures and the transparency of selection. Despite the systematic advantages of this approach, the sole use of the Web of Science database as a source of literature may have resulted in the omission of other important studies. Particularly when it comes to research at the intersection of K-12 education and physical education (3, 4), databases such as PubMed, ERIC, and Scopus may provide valuable literature that is not included by Web of Science (5). Therefore, the limitations of literature selection may affect the comprehensiveness of the review, as relevant studies from these other databases could have contributed further insights to the analysis (Figure 1).

The application of the theoretical framework in this review focuses on self-determination theory (SDT) and constructivism theory. While these theories offer foundational guidance for blended learning research, their implementation remains relatively superficial, primarily confined to conceptual descriptions rather than concrete pedagogical applications (6, 7). The limitations manifest in two aspects: (1) insufficient exploration of how these theories can be systematically operationalized to enhance instructional effectiveness in blended learning environments, particularly in physical education contexts (8); (2) absence of discipline-specific adaptations addressing students' kinesthetic characteristics and motor learning needs, with limited practical strategies or case demonstrations. Future research should adopt diversified theoretical frameworks with complementary strengths. For instance, social learning theory could enhance collaborative skill acquisition through video-modeled demonstrations and peer feedback mechanisms in blended PE modules (9), while metacognitive theory could improve self-regulated learning via reflective journals and goal-setting scaffolds within digital learning platforms. Such multidimensional theoretical integration would better address the psychomotor, cognitive, and affective dimensions of physical education.

In terms of participant groups, Wang et al. mainly focused on undergraduate students in their study; however, the adaptability and self-regulation abilities of K-12 students in blended learning are usually poor and fail to be fully reflected in the study. Existing research highlights unique challenges at the K-12 level: for instance, Miller (10) found that middle school students struggled with time management and digital literacy in hybrid physical education courses, requiring structured scaffolding from teachers. Similarly, Thomas et al. (11) reported that elementary students exhibited lower engagement in asynchronous video-based skill practice compared to face-to-face instruction. K-12 students face systemic challenges in technology adaptation and self-management, particularly in physical education where motor skill acquisition depends on real-time feedback (12). While the PE teacher's role in blended learning is equally critical—such as designing multimodal feedback systems and bridging online theory with offline practice—these groups have been less studied, limiting the assessment of the full effects of blended learning (12). Future research should further explore the involvement of K-12 student and teacher groups, building preliminary frameworks like Lindberg et al. (13) “blended PE pedagogy” that integrates wearable sensors for skill analytics in secondary schools. Priorities include examining how teachers adapt to the new teaching model, especially through competency-based training programs to enhance technology acceptance, as well as evidence-based strategies for improving their instructional design ability in blended learning contexts.

The diversity of learning tools is an important factor in the effect of blended learning, but the review by Wang et al. mainly mentions the application of tools such as online learning platforms and learning software. While these tools are foundational, their standardized design often neglects the psychomotor and affective demands inherent to physical education. Notably, the article fails to discuss how tools align with students' psychological needs (e.g., autonomy in skill progression) or mitigate cognitive load through pedagogical design (14). Emerging technologies like virtual reality (VR) and gamified learning offer targeted solutions to these gaps. For instance, VR environments can simulate sport-specific scenarios (e.g., Table tennis tac-tical simulations) while providing real-time biomechanical feedback to reduce extraneous cognitive load (15). Future research should prioritize contextualized tool design in physical education, exploring how adaptive technologies like VR can bridge theory-practice gaps in complex motor skill acquisition, or how gamification mechanics might be tailored to satisfy competence needs within cognitive load theory frameworks.

The evaluation methods summarized by Wang et al. are mainly based on questionnaires and tests. These quantitative methods can collect certain learning data, but fail to fully cover the non-cognitive factors such as emotion and motivation that may be generated by students in the process of blended learning. Qualitative assessment methods such as reflective logs and observation notes mentioned in this article have not been widely used, showing the uniformity of assessment methods in this field. Blended learning as a multi-dimensional teaching model, its effect is not only reflected in academic performance, but also in students' emotional experience, learning motivation, social interaction and other aspects. Therefore, future research should adopt a mixed approach (such as qualitative + quantitative) to comprehensively evaluate the effects of blended learning, especially at the affective and cognitive levels (16).

In general, the review by Wang et al. provides a valuable reference for the application of blended learning in physical education, but it also exposes some limitations, such as the limitation of literature sources, the uniqueness of the application of theoretical framework, the inadequacy of participant groups, and the bias of evaluation methods. Future research should focus on the following aspects: first, expanding the sources of literature, especially considering more groups including K-12 education and teachers; The second is to strengthen the application of multiple theoretical frameworks, especially the combination of educational psychology and social learning theory; The third is to adopt more diverse evaluation methods to comprehensively measure the multi-dimensional impact of blended learning.
